# Sitting with others: mental health self-help groups in northern Ghana

**DOI:** 10.1186/1752-4458-6-1

**Published:** 2012-03-21

**Authors:** Alex Cohen, Shoba Raja, Chris Underhill, Badimak Peter Yaro, Adam Yahaya Dokurugu, Mary De Silva, Vikram Patel

**Affiliations:** 1 Mental Health in the Faculty of Epidemiology and Population Health at the London School of Hygiene & Tropical Medicine, London, UK; 2 BasicNeeds, Banaswadi, Bangalore, India; 3 BasicNeeds, Lemington Spa, UK; 4 BasicNeeds, Accra, Ghana; 5 BasicNeeds, Tamale, Ghana

## Abstract

**Background:**

Over the past four decades, there has been increasing interest in Self-Help Groups, by mental health services users and caregivers, alike. Research in high-income countries suggests that participation in SHGs is associated with decreased use of inpatient facilities, improved social functioning among service users, and decreased caregiver burden. The formation of SHGs has become an important component of mental health programmes operated by non-governmental organisations (NGOs) in low-income countries. However, there has been relatively little research examining the benefits of SHGs in this context.

**Methods:**

Qualitative research with 18 SHGs, five local non-governmental organisations, community mental health nurses, administrators in Ghana Health Services, and discussions with BasicNeeds staff.

**Results:**

SHGs have the potential to serve as key components of community mental health programmes in low-resource settings. The strongest evidence concerns how SHGs provide a range of supports, e.g., social, financial, and practical, to service users and caregivers. The groups also appear to foster greater acceptance of service users by their families and by communities at large. Membership in SHGs appears to be associated with more consistent treatment and better outcomes for those who are ill.

**Discussion:**

This study highlights the need for longitudinal qualitative and quantitative evaluations of the effect of SHGs on clinical, social and economic outcomes of service users and their carers.

**Conclusions:**

The organisation of SHGs appears to be associated with positive outcomes for service users and caregivers. However, there is a need to better understand how SHGs operate and the challenges they face.

## Background

Mental health Self-help Groups (SHGs) may be defined as, "any mutual support oriented initiative directed by people with mental illness or their family members" [1]. SHGs may have different objectives: while some may be primarily concerned with the provision of peer support, others may devote their efforts toward changing public policies and, more broadly, changing public attitudes. Still others may focus on self-empowerment, including monitoring and critiquing the mental health services they are receiving [2,3].

Over the past four decades, there has been increasing interest in SHGs by mental health services users and care-givers alike [1]. With this has come research demonstrating the benefits of participation in SHGs [4,5]. Research in high-income countries has demonstrated a number of benefits of participation in SHGs. For example, participation in an SHG for clients of the South Verona (Italy) Community Psychiatric Service was associated with decreased use of inpatient facilities [6]. In the US, a 12-week family and peer-based education programme was found to be associated with decreased levels of worry and depression, as well as increased feelings of empowerment [7,8]. In the Netherlands, a randomised-controlled trial demonstrated that peer-support groups for people with psychosis had a positive effect on social support and social networks [9]. In Hong Kong, research demonstrated that family SHGs were associated with improved patient functioning and decreased caregiver burden [10,11].

The formation of SHGs has become an important component of mental health programmes operated by non-governmental organisations (NGOs) in low-income countries [2]. However, there has been relatively little research on the topic in these settings. Perhaps the best demonstration of the efficacy of SHGs comes from the work of Chatterjee et al. in rural India [12,13]. During a four-year follow-up of a cohort of persons with severe mental disorders, participation in SHGs was an independent predictor of improved social functioning, e.g., voting, attending festivals, and working.

BasicNeeds - an NGO that has recently or is currently working in India, Sri Lanka, Ghana, Uganda, Tanzania, Kenya, Nepal, Vietnam and Laos PDR - seeks to implement a social and economic development approach to mental health care. The basic tenet of this strategy, which is called the Model for Mental Health and Development, is that long-term recoveries can only be achieved if programmes address the multiple challenges (e.g., clinical, social, and economic) faced by individuals with mental disorders and epilepsy [14]. Thus, BasicNeeds seeks to help individuals and their families access treatment services, to reduce stigma, and to provide livelihood programmes that address the negative economic consequences of mental disorders and epilepsy.

There is limited documentation of how mental health services in low-income countries function in practice [15], and this lack of knowledge imposes a serious challenge to the goal of scaling up effective mental health services [16,17]. There is also a comparative lack of information about SHGs, even though this psychosocial intervention is a central feature of the work of BasicNeeds and other NGOs, e.g. CBM [2], that operate mental health programmes in low-income countries.

The exploratory research that is in the focus of this paper was one aspect of the Case Studies Project at the London School of Hygiene and Tropical Medicine. The project was established with the objective of developing a methodology that would provide a means, when clinical trials are not feasible, by which to systematically document, compare, and assess Community Mental Health service delivery models [18]. The SHGs organised by BasicNeeds in northern Ghana were chosen as one focus of the project because of the need to augment our understanding of how SHGs may contribute to the clinical, social, and economic well-being of service users and their families.

## Methods

The findings are based on two site visits that the first author (AC, an anthropologist with experience in ethnographic research and analysis of qualitative data and who has written and edited a number of case studies of mental health programs in low-income settings [18-20]) made site visits to three regions of northern Ghana over a total of 18 days in 2008 and 2009. During these visits, AC met with representatives of five local non-governmental organisations that worked with BasicNeeds, spoke with community mental health nurses, met with District and Regional administrators in the Ghana Health Services, and held discussions with BasicNeeds staff. Information gleaned from these discussions and conversations was documented by AC in fieldnotes. In addition, AC held meetings with SHGs in the following districts and regions: Bongo, Upper East (1), Lawra, Upper West (1), Walewale, Northern (11), Yendi, Northern (3), and Tamale, Northern (2); he also met with members of two district associations of users and caregivers. During these meetings, AC inquired about and collected information on such topics as the number of families in each SHG, why people joined, how many members took advantage of a loan programme, and the perceived benefits of membership in the groups. The meetings were conducted in local languages; a BasicNeeds staff member (AYD) translated for AC. Discussions and conversations with BasicNeeds staff, representatives of local NGOs, Community Psychiatric Nurses, and Ghana Health Service officials were conducted in English.

Observations about the settings in which the SHGs functioned and information about the BasicNeeds programme, in general, and the SHGs, in particular, were documented by AC in fieldnotes that, together with the fieldnotes about the meetings with the SHGs and key informants, were then converted into narrative accounts. Qualitative data analysis software (HyperResearch, version 2.8.3) was used to extract information and identify themes in the narrative accounts. Supplementary information about BasicNeeds and mental health services in Ghana was collected from documentary evidence that AC obtained from the BasicNeeds office in Tamale, Northern Ghana and the BasicNeeds website http://www.basicneeds.org/.

## Results

### Context

The BasicNeeds Mental Health and Development programme operates in the three administrative regions (Northern, Upper East, and Upper West) of northern Ghana, an area that encompasses about 98,000 square kilometers or more than 45% of the landmass of country [21]. There are no psychiatric facilities or psychiatrists in the regions. A handful of Community Psychiatric Nurses - who are based at District Hospitals operated by Ghana Health Services - provide the only biomedical treatments for mental disorders and epilepsy. BasicNeeds does not provide any treatment services. Rather, it facilitates access to what is offered by the Ghana Health Services, works to empower people to advocate on their own behalf, and assists with the economic development of those affected by the consequences of mental disorders and epilepsy. The programme works toward these goals through activities that include: funding of regular visits of psychiatrists to the three northern regions, supporting the work of Community Psychiatric Nurses, providing training programmes for health staff, working with the local NGOs that organize and support the SHGS, and operating a loan programme that provides credit to service users and caregivers.

### Establishment of the SHGs

The BasicNeeds programme was established in northern Ghana in 2002, but SHGs were only organised in 2006, after individuals with mental disorders and epilepsy and their caregivers stated that belonging to groups would offer them the opportunity to discuss issues of mutual interest and to provide support to each other.

The first groups were large and consisted of people who came from widely scattered communities. Travel to meetings of the SHGs proved to be a problem. Therefore, between 2007 and 2009, the SHGs were re-organised so that groups would be based, as far as possible, in single communities so that it would be easier for members to attend meetings. Thus, the SHGs would be more sustainable. As of April 2009, BasicNeeds was supporting 71 SHGs in the Northern Region, 34 in the Upper East, and 16 in the Upper West. According to figures from the BasicNeeds database, SHGs have, on average, 20 families. As of 31 December 2010, BasicNeeds reports that almost 18,000 service users participate in its programmes in northern Ghana. The diagnostic categories of participants are summarized in Table 1. In general, about one-third of service users in the programme join SHGs; approximately half of SHG membership is comprised of caregivers [22].

**Table 1:** All service users who have participated in programme of BasicNeeds, northern Ghana, 2002 - 2010

DIAGNOSIS	ADULTS Age 18+		ADOLESECENTS Age 14-17		CHILDREN Age 13 & below		Total N (%)
	
	M	F	M	F	M	F	
**Epilepsy**	3028	2496	607	525	1851	1425	**9932 (55.5)**

**Common Mental Disorders**							

Anxiety	92	113	2	11	9	9	236

Depression	153	339	4	13	1	3	513

**Sub-total**	**245**	**452**	**6**	**24**	**10**	**12**	**749 (4.2)**

**Severe Mental Disorders**							

Schizophrenia	802	738	23	24	18	17	1622

Unspecified Psychosis	1764	1379	82	64	57	53	3399

Bipolar Disorder	2	8	1	0	0	0	11

Depression with Psychotic Symptoms	39	59	0	3	5	3	109

**Sub-total**	**2607**	**2184**	**106**	**91**	**80**	**73**	**5141 (28.7)**

Other Mental Disorders*	892	698	48	30	177	144	**1989 (11.1)**

**Diagnosis not known**	41	26	1	3	5	3	**79 (0.4)**

**Sub-total** **All Service Users since beginning of the programme**	6813	5856	768	673	2123	1657	**17890 (100.0)**

No longer in programme**	737	581	101	65	241	181	**1906**

**TOTAL** **Active Service Users**	6076	5275	667	608	1882	1476	**15984**

*Includes dementia, headache, substance abuse, autism etc.

**Those who have stopped participating or cannot be traced

Only active mental health service users and their families are eligible to join SHGs.

### Increased membership

The SHGs were attracting increasing numbers of members. Of the 10 groups from which information was collected, seven reported large increases, two reported that more families were about to join, and only one reported a decrease in membership (Table 2). Increases in membership were generally attributed to the following reasons. First, members of one SHG believed the increase was the result of families recognizing that SHG membership was associated with consistent treatment and improved outcomes (see below). For example, a member of one group reported how people in the community now come to group members to ask advice about how to cope with ill family members. Second, membership offers the possibility of obtaining loans (see below). Although access to treatment and medication were always mentioned as being of paramount importance, the significance of access to credit cannot be overstated. For example, one of the community psychiatric nurses spoke of a man who had kept his daughter out of treatment for five years and only joined an SHG because of the possibility of obtaining a loan. While this was not at all typical of the reasons people gave for joining SHGs, it is in line with how members spoke of their need for credit.

**Table 2:** Increased membership in SHGs (2006-2009)

SHGs	Original # of families	Current # of families	Increase N (%)
Kumlan-Fong	14	18	4 (29)

Tinguri	8	14	6 (75)

Yendi 1	12	19	7 (58)

Yendi 2	22	32	10 (45)

Nakpanzoo	6	20	14 (233)

Nasia, Tiya, & Janga	18	42	24 (133)

Tamale	10	35	25 (250)

### Management of the SHGs

Most SHGs meet once a month. If a group has members from more than one community, a meeting is held for the entire group and separate, smaller meetings are held for members who live in the different communities.

Most SHGs collect dues. These are usually .10 or .20 Cedis (≈0.06-0.12USD) per month, but some groups have dues of 1 Cedi per month (≈0.67USD). The payment of dues is not strictly enforced and contributions are frequently seasonal, i.e., during harvest season the rates of payment are high and are much lower in April through June when northern Ghana is prone to food shortages.

Funds from dues are often used to support families in times of acute financial needs, e.g., during times of bereavement, births, and weddings. In addition, funds from dues may be used to help families that are in need of food (because of seasonal food shortages) or medication (because supplies at the District Hospitals tend to be erratic and families may be forced to fill and pay for prescriptions at pharmacies). The funds generated from dues should not be considered micro-credit since they are used primarily to pay for transportation to collect medications at district hospitals or to help families in dire need. In general, dues are not used as investments to support economic activities - although members of one SHG did report that they were planning to use the dues to buy grain in anticipation of seasonal food shortages. If the season did not bring shortages, the group would sell the grain for a profit.

Most SHGs have bank accounts in which the money collected as dues or repayments of loans is kept (see below).

### Loan programme

One goal of BasicNeeds is to help people with mental disorders and epilepsy become productive members of the communities in which they live, and, for that reason, providing access to credit is a central element of its work with SHGs. In general, BasicNeeds gives funds to local NGOs that, in turn, dispense loans to group members. The process of applying for loans is as follows. First, an applicant must be a member of an SHG and attest that the ill family member is receiving treatment. Stabilized users who are members are also eligible to apply. Individual members apply to their SHGs and indicate how the loan will be used (e.g., petty trading, farming, selling prepared food, processing shea nuts, and buying and selling grain) and how much is needed. The applications are compiled by the SHGs that, in turn, submit the requests to the local NGO that oversees the programme. The NGO then reviews the applications and accepts those that are considered reasonable. The NGO may adjust the amount of the loan according to its assessment of the proposal, and may only approve the loan for a lower amount. The NGO then disburses the loans to the individual applicants.

Loans are generally in the range of 100 to 200 Cedis (≈60 to 120 USD) but some are as much as 2,000 Cedis (≈1,200 USD). The size of these loans far exceeds the amounts that SHGs could raise through dues. Moreover, the amounts are quite large in comparison to average incomes. According to the United Nations Statistics Division, the per capita gross national income for Ghana was 698USD in 2008 http://data.un.org/CountryProfile.aspx?crName=Ghana. However, is much poorer than the country as a whole, and per capita incomes in the Upper East and Upper West Regions may be as little as 25% of per capita incomes in Greater Accra (http://www.statsghana.gov.gh/docfiles/glss5_report.pdf).

Initial loans to individuals are made interest free, but subsequent loans carry interest charges of 12.5% per year. Ideally, this system is intended to generate enough funds to create on-going, revolving loan programmes that are managed and run by the SHGs with support and supervision from a local NGO and a District Association.

There is a range of experiences in regard to taking out and repaying loans. Virtually all SHGs members reported that they had been given the opportunity to secure loans and many members had taken advantage of that opportunity. However, repayment rates and the proportion of members who took out loans fluctuated substantially across the groups. For example, members of one SHG reported that everyone had taken out loans and that, so far, one-third of the loans had been repaid, while the members of another group reported that floods and crop failure meant that it was not possible to repay any of their loans.

SHG members mentioned that the loans, and the profits earned as a result of them, also went to buy medications when there were lapses in supplies at the district hospital and the money saved from collected dues was not sufficient to cover the cost of medication purchases at local pharmacies. However, it must be noted that the use of loans for the purchase of medications or the care of a service user can be a serious problem. For example, one informant reported that he had received a loan to buy shea nuts but had been forced to spend the loan on the care of a service user in his family who had relapsed. As a result, the man defaulted on the loan.

The loan programme, at least how it was conducted among the SHGs visited in April 2009, faced four challenges. First, SHG members reported little contact with the local NGO that was administering the programme. This was, at least in part, the result of the NGO not being able to hire more than one field officer to work with several dozen groups that were scattered across a large area. Second, record keeping has been less than adequate, e.g., it was not possible to determine the proportion of families that have received loans, the rates of repayment, and the number of families that have taken out multiple loans. As a result, it is difficult to assess whether the loan programme is achieving its primary goal: the economic betterment of families and their ill members. Third, there is a question of whether the loan programme is sustainable, i.e., whether repayment rates and the interest earned on loans will be sufficient to keep the loan programme solvent and able to provide loans to increasing numbers of SHG members. For example, the BasicNeeds loan programme provides one-time or occasional infusions of funds rather than on-going access to credit. Thus, if the members of an SHG all experience crop failure (which is a real threat in northern Ghana), that group will not have access to credit until BasicNeeds can provide another infusion of funds. Fourth, it is critical to assess whether the benefits, if any, of the loan programme have been felt by service users. That service users might not benefit, at least in a material way, was a possibility raised by an NGO worker in the Upper East who was concerned that some families were not using their profits to improve the lives of ill members.

### Support

SHGs provide a range of social and some financial support to members. For example, if a member misses a meeting, someone will visit their home to see if they are in need of help. One man stated that group members look after ill members from other families if they are found wandering. When a woman in one SHG had a child, other members drew water, collected wood, and cooked for her family. Members of another group reported that they had helped each other plaster and floor their homes. Members of yet another group reported how, in the event of a death, they would participate in bereavement customs, as well as help with cooking, grinding maize, and fetching water for the bereaved. Two SHGs had devised plans for families to take turns caring for ill members so that people could tend their farms during the rainy season.

Support also comes in the form of financial aid, especially when families are in need of food or medication, or at times of bereavement, the birth of a child, and weddings. Funds for this support come from membership dues.

Other support is related to the burden of care-giving. For example, if someone cannot go to the hospital to collect a prescription, another member of the group will get it for her/him. Members encourage each other to be certain that users take their medication. Members also encourage each other to seek the advice and help of a community psychiatric nurse when treatments are not effective. More generally, support comes in the form of having an opportunity to talk about the difficulties of care-giving. SHG members urge each another to have patience and tolerance toward ill family members, even when caregiving is emotionally demanding. One individual stated that when the ill family member does not sleep, the caregiver will not be able to sleep - and when the ill family member's condition is not stable, it is difficult for the caregiver to concentrate on work. Another SHG member spoke of how difficult caregiving was when the ill family member had poor self-hygiene, could not talk, or refused to eat. A third described how it is difficult to leave ill members alone. And a fourth spoke of how she must, at times, leave social gatherings because her son is having a seizure, and how this leaves her feeling isolated. Individuals in two SHGs reported that anti-seizure medication, probably carbamazepine, caused an increase in the appetite of persons with epilepsy, and that this was a particular problem during seasonal food shortages. Two others spoke of being frustrated by the lack of their children's improvement: a woman spoke of her daughter whose seizures had stopped, but who did not look "normal" and who continues to cry, scream, and urinate on herself, while a man reported that although his child was on medication, there had been no significant change in his condition. Last, a woman spoke of how, with treatment, her daughter had been stabilized and now wants to go back to school, but that the family's lack of money makes this impossible.

The importance of being able to share experiences and advice cannot be overstated. Perhaps the most telling comment in this regard was offered by a woman who commented that before the formation of the SHGs, everyone thought they were alone, and now they know that is not true.

### Promotion of biomedical treatment

Reports suggest that SHG membership is associated with positive changes in the treatment of mental disorders and epilepsy in that the families in the groups appear to be effective in encouraging ill service users to take medications, while also supporting fellow caregivers to monitor the care of their ill family members. For example, one woman related how SHG members had told her about the services at the district hospital, how she followed their advice to seek care, and how her daughter is now taking medication. Similarly, a man related that an SHG member in his community had introduced him to the psychiatric unit at the district hospital and how, as a result, he had started treatment and has not had a seizure for two years.

Other members of the SHGs spoke, too, of positive outcomes as a result of biomedical treatment, access to which was facilitated by BasicNeeds and promoted by the SHGs. For example, one informant related how she managed to convince her daughter to take medications and how her daughter's self-care had improved. As a result, her daughter had gone back to school and had learned to read and write. Another woman had a similar story about a daughter who, with medication, had been able to return to school. A third informant spoke of a son who, with treatment, had also been able to return to school and to play football. And a fourth informant spoke of a son who, with treatment, no longer strips naked and runs away. Two service users spoke of their positive experiences with treatment. An older woman stated that since she began to take medication she no longer stripped naked and ran into the bush. A young man recounted how he had sought care at several hospitals because he was sad and had trouble sleeping, but that none of the treatments had been effective. However, he was feeling better now that he was taking medication and under the care of the Community Psychiatric Nurse at the District Hospital.

One change that seems to have come about as a result of effective biomedical treatment is that some families are no longer seeking care with traditional healers. This change has come about for two reasons. First, people recognize that biomedical treatments are effective, while those offered by traditional healers are not. Second, traditional healers, who demand payment in goats or domesticated fowl, are expensive, e.g., one woman reported that going to healers had impoverished her, and others confirmed that healers were very costly. Nor were the healers necessarily supportive, e.g., one healer told a mother that her ill child was not a human being, while another healer told a mother that her child was a snake. However, some experiences with healers were positive. Two SHG members stated that they were combining traditional and biomedical treatments. These are anecdotes from a relatively small sample of individuals, but they are consistent with other reports about traditional healers in northern Ghana [23,24].

### Increased social inclusion

SHGs appear to be responsible for positive social changes within families and communities. One often mentioned change was that people no longer believed that epilepsy was contagious. This has meant that people are now less likely to run away, and may even be willing to help when someone experiences a seizure. With treatment and the control of symptoms, as well as greater understanding of epilepsy and mental disorders, some ill children have been allowed to return to school, parents do not worry about them so much, younger children are beginning to respect older siblings who are ill, and caregivers are more accepting and treat ill family members with dignity. It has also meant that families are more likely to allow children with epilepsy to venture outside on their own. Physical care has changed, too. For example, two informants spoke of how they now follow the need to hold and support someone who is experiencing a seizure.

The power of the SHGs to reduce social exclusion can be encapsulated in the following comments. One user stated that when his condition was severe he was isolated, but now that he was getting treatment and his condition was under control, he was not isolated and that he was now allowed to lead community prayers. In one meeting, AC was told that ill persons were once stigmatized by their own families, that they were made to drink water from different vessels and to sit apart at meals - but that was no longer the case. And, finally, a man related that he had a history of stripping naked publically, but with treatment he was now allowed to sit with others. Thus, in an immediate and personal way, the SHGs contribute to a process through which individuals with epilepsy or mental illness are re-integrated into family and community life.

## Discussion

The findings reported here, which mirror results of studies in high-income countries, suggest that SHGs are of benefit in terms of increasing social inclusion, providing social support to care-givers and promoting biomedical treatments for mental disorders and epilepsy. Thus, SHGs have the potential to serve as key components of community mental health programmes in low-resource settings. The interviews with SHG members also indicate that the BasicNeeds loan programme provides access to critically needed credit that would not otherwise be available. At the same time, and in view of research suggesting that SHGs, and micro-credit schemes, in particular, may not be as beneficial as often assumed [25-27], the findings of this exploratory study must be viewed with caution.

Perhaps the most important result of this exploratory study is the identification of questions in need of further investigation. Furthermore, one must take into consideration that the organisation, implementation, and management of SHGs represent a complex intervention [28]. As such, evaluating the effectiveness of SHGs to improve the quality of lives of service users and caregivers requires multi-dimensional research that examines clinical, social, and functional outcomes, the economic benefits of memberships, and the feasibility and acceptability of SHGs in specific local settings. Four suggestions for such research follow.

First, the lack of longitudinal, quantitative data makes it difficult to assess the extent to which participation in the SHGs has an influence on mental health outcomes. For example, is participation in SHGs associated with improved clinical, social, and functional outcomes of service users and caregivers? This question would require the assessment of the clinical, social, and functional statuses of service users and family members at the time they join an SHG and then reassessment 6 or 12 months later to determine the extent to which service users and family members had experienced improvement in these three domains. It might also be of interest to examine the association between frequency of participation in SHG meetings and clinical, social, and functional outcomes.

Second, a lack of data about loan repayment rates and changes in the economic status of individuals and families makes it difficult to assess the extent to which SHG members had been able to use the loans successfully and whether participation in the programme was, in fact, associated with improved economic status and, more generally, well-being. A better understanding of the effects of the loan programme would require data on the proportion of SHG members who had received initial and subsequent loans, the rates at which loans were repaid successfully, and if the material qualities of their lives had improved.

Third, the anecdotal evidence cited above strongly suggests that the formation of SHGs was associated with increased social support for family members and greater social inclusion of service users. While there is little reason to deny the reliability of these reports, longitudinal, qualitative research would provide insights about the extent and types of support provided, as well as the extent to which SHGs can bring about changes in family and community attitudes to individuals with mental disorders or epilepsy.

Fourth, further investigation is needed to determine the possible role of traditional healers in the process of scaling up community mental health services. The preliminary findings reported above, suggest that we may not have a detailed understanding of why people seek the care of healers or under what circumstances people with mental disorders or epilepsy stop seeking such care or seek biomedical and traditional treatments simultaneously.

## Conclusions

The findings from this exploratory research suggest that SHGs have the potential to serve as key components of community mental health programmes in low-resource settings. The strongest evidence concerns how SHGs provide a range of supports, e.g., social, financial, and practical, to service users and caregivers. The SHGs also appear to foster greater acceptance of service users by their families and by communities at large. Furthermore, membership in SHGs appears to be associated with more consistent treatment and better outcomes for those who are ill. The evidence about engagement with the loan programme was also positive in that many SHG members had taken advantage of not one, but several opportunities to obtain credit to engage in economic activities that would not otherwise have been available to them.

At the same time, one must acknowledge several limitations to this study, which are, most importantly: a lack of quantitative data on financial and mental health outcomes, reliance on anecdotal evidence, and non-random sampling of SHG members.

One should not, however, conclude that the limitations of this exploratory research negate its findings. There is comparatively little research about SHGs and this study has shown preliminary evidence of a range of positive effects in a number of domains, and points the way to further research using more robust study designs. This is important so that we might better understand how SHGs function, the challenges they face, and the potential benefits offered by SHG membership. Ultimately, the questions we have posed are put forward so that we can better examine the effects of membership in SHGs, augment those elements that are positive and avoid those that are negative, and better determine the role of SHGs in the process of scaling up mental health interventions and reducing the treatment gap in low-resourced settings.

## Competing interests

The authors declare that they have no competing interests.

## Authors' contributions

AC designed the study, conducted the fieldwork, conducted the data analyses, and drafted the initial manuscript. SR made substantial contributions to the conception of the study and reviewed the manuscript for important intellectual content. CU and BPY reviewed the manuscript for important intellectual content. AYD assisted in the collection of data and reviewed the manuscript of important intellectual content. MDS and VP were involved in designing the study, and drafting the manuscript and revising it critically. All authors have read and approved the final manuscript.
